# Squeezing Out Secrets: How Pressure Reveals Structural Dynamics Across Whole Proteomes

**DOI:** 10.1063/4.0000997

**Published:** 2025-10-27

**Authors:** Richard E Gillilan, Haley Moran, Evelyn Patterson, Shreya Palakurthi, Edgar Manriquez-Sandoval, Stephen Fried

**Affiliations:** 1 Center for High Energy X-ray Sciences, Cornell University, Ithaca, NY, USA; 2 Department of Chemistry, Johns Hopkins University, Baltimore, Md, USA; 3 Department of Biology, Johns Hopkins University, Baltimore, Md, USA; 4 Department of Biomedical Engineering, Johns Hopkins University, Baltimore, Md, USA; 5 Department of Biophysics, Johns Hopkins University, Baltimore, Md, USA

## Abstract

The effects of hydrostatic pressure on biomolecules are seemingly paradoxical. Rather than simply shrinking in size, biomolecules often reduce their effective volume using conformational changes that expose natural voids to the solvent. Due to hydrogen bond networks, bulk water itself is a very open structure that when disrupted, can collapse, effectively reducing its own volume. This is most evident in the well-known phenomenon of electrostriction, in which highly charged ions effectively compress surrounding layers of bulk water. Thus, both biomolecules and surrounding layers of water and ions can respond to pressure in potentially revealing ways. For this reason, hydrostatic pressure has recently proven a valuable means of populating physiologically important, but normally invisible conformational states (Wang J, et al. (2023) PNAS 120:e2215556120). Until now, we have had no way to tell, in the vast sea of biomolecules, which are pressure sensitive. To better identify such proteins and to help understand their structural behavior and physiological role, we introduce a novel structural technique: high-pressure limited proteolysis (HiP- LiP), a powerful new tool for detecting subtle conformational changes in response to pressure on a proteome-wide scale (Moran HM, et al. (2024) PRX Life 2:033011). By mapping residue-level changes to both experimental and predicted structures, we find that pressure-sensitive regions correlate with active sites and functionally relevant structural dynamics, particularly involving highly charged species. These maps are a rich source of new targets for structural and biophysical studies, possibly revealing unknown binding partners and associated dynamics under both normal and extreme conditions. This talk will discuss the methodology of HiP-LiP, analyze proteome-wide tends, and present multiple structural examples.

